# Induction of mucosal immunity by pulmonary administration of a cell-targeting nanoparticle

**DOI:** 10.1080/10717544.2021.1955040

**Published:** 2021-07-22

**Authors:** Tomoaki Kurosaki, Yuki Katafuchi, Junya Hashizume, Hitomi Harasawa, Hiroo Nakagawa, Mikiro Nakashima, Tadahiro Nakamura, Chikamasa Yamashita, Hitoshi Sasaki, Yukinobu Kodama

**Affiliations:** a Department of Hospital Pharmacy, Nagasaki University Hospital, Nagasaki, Japan; b Graduate School of Biomedical Sciences, Nagasaki University, Nagasaki, Japan; c Faculty of Pharmaceutical Sciences, Tokyo University of Science, Chiba, Japan

**Keywords:** Vaccine, pulmonary administration, nanoparticles, γ-polyglutamic acid, mucosal immunity

## Abstract

We previously found that a nanoparticle constructed with an antigen, benzalkonium chloride (BK) and γ-polyglutamic acid (γ-PGA) showed high Th1 and Th2-type immune induction after subcutaneous administration. For prophylaxis of respiratory infections, however, mucosal immunity should be induced. In this study, we investigated the effect of pulmonary administration of a nanoparticle comprising ovalbumin (OVA) as a model antigen, BK, and γ-PGA on induction of mucosal immunity in the lungs and serum. The complex was strongly taken up by RAW264.7 and DC2.4cells. After pulmonary administration, lung retention was longer for the OVA/BK/γ-PGA complex than for OVA alone. OVA-specific serum immunoglobulin (Ig)G was highly induced by the complex. High IgG and IgA levels were also induced in the bronchoalveolar lavage fluid, and *in vivo* toxicities were not observed. In conclusion, we effectively and safely induced mucosal immunity by pulmonary administration of an OVA/BK/γ-PGA complex.

## 1. Introduction

Acute respiratory infections almost certainly are the leading cause of death among children <5-years-old in developing countries (Williams et al., 2002). Several vaccines, such as those for influenza, pertussis, and pneumococcus, have been developed for respiratory infections (White, 1988; Pittman, 1991; Chen et al., 2020). Most of those vaccines are administered by the intramuscular and subcutaneous routes, which could effectively induce serum immunoglobulin (Ig)G. However, other routes are more useful to stimulate mucosal immunity for preventing acute respiratory infections. Mucosal administrations, such as intranasal and pulmonary, have been reported to effectively induce mucosal IgA and prevent respiratory infections (Giri et al., 2005; Ainai et al., 2017).

On the other hand, vaccines for intranasal and pulmonary administration should not contain adjuvants that have been reported to cause inflammatory reactions and ulceration at the administration site (Tamura et al., 1988; McKee et al., 2007). Another approach for improving the efficacy of vaccines without adjuvants is to develop a system that can effectively deliver vaccines into mucosal antigen-presenting cells (APCs).

In a previous study, we developed a novel vaccine delivery vector constructed with an antigen, benzalkonium chloride (BK) and γ-polyglutamic acid (γ-PGA). A complex comprising ovalbumin (OVA) as a model antigen, BK and γ-PGA (OVA/BK/γ-PGA complex) effectively delivered OVA into dendritic cells and improved OVA-specific serum IgG induction after subcutaneous administration into mice (Kurosaki et al., 2012). The aim of the present study was to investigate the effect of pulmonary administration of an OVA/BK/γ-PGA complex on the induction of mucosal immunity in the lungs and serum.

## 2. Materials and methods

### 2.1. Chemicals

OVA was purchased from Sigma-Aldrich (St. Louis, MO, USA). BK was obtained from Nacalai Tesque, Inc. (Kyoto, Japan). The γ-PGA was provided by Yakult Pharmaceutical Industry Co., Ltd. (Tokyo, Japan). Fluorescein isothiocyanate-labelled OVA (FITC-OVA) and Alexa Fluor 647-labeled OVA (Alexa647-OVA) were obtained from Invitrogen (Carls, CA, USA). Fetal bovine serum (FBS) was purchased from Biological Industries Ltd. (Kibbutz Beit Haemek, Israel). OPTI-MEM I was obtained from GIBCO BRL (Grand Island, NY, USA), and a premix antibiotics solution containing penicillin, streptomycin, and L-glutamine were obtained from Wako Pure Chemical Industries, Ltd. (Osaka, Japan). Dulbecco’s Modified Eagle Medium (DMEM) and RPMI1640 medium were obtained from Nissui Pharmaceutical Co., Ltd. (Tokyo, Japan).

### 2.2. Complex preparation

Previously, we constructed an OVA/BK/γ-PGA complex at a weight ratio of 1:0.2:0.2 (Kurosaki et al., 2012). To prepare the OVA/BK complex, an appropriate amount of BK solution (pH 5.0) dissolved in 5% glucose was mixed with OVA solution (pH 7.0) dissolved in 5% glucose and left for 15 minutes at 4 °C. To coat the OVA/BK complex with γ-PGA, a γ-PGA solution (pH 7.0) dissolved in 5% glucose was added to the OVA/BK complex and left for a further 15 min at 4 °C. The particle size and ζ-potential of each complex were measured by using a Zetasiser Nano ZS (Malvern Instruments, Ltd., Malvern, UK).

### 2.3. Cells

Mouse macrophage cell line RAW264.7 and dendritic cell line DC2.4 were used. RAW264.7 cells were grown in DMEM supplemented with 10% FBS and antibiotics. DC2.4 cells were grown in RPMI1640 medium supplemented with 10% FBS, antibiotics, 1 mM non-essential amino acids, and 1 nM 2-mercaptoethanol. These cells were assessed under a humidified atmosphere of 5% CO_2_ in air at 37 °C.

### 2.4. *In vitro* cellular uptake experiment

RAW264.7 cells and DC2.4 cells were plated onto 24-well plates (Corning, NY, USA) at a density of 2.0 × 10^4^ cells/well and cultivated in 500 µL of culture medium. After 24-h preincubation, the medium was replaced with OPTI-MEM I medium, and the cells were incubated with 5 µg FITC-OVA and the complex containing 5 µg FITC-OVA for 2 h. After incubation, those cells were washed with PBS and observed under a fluorescent microscope (BIOREVO BZ-9000; Keyence Co., Osaka, Japan). After observation, those cells were lysed in 300 μL of lysis buffer (pH 7.8 and 0.1 M Tris/HCl buffer containing 0.05% Triton X-100 and 2 mM EDTA). The lysates were placed into 96-well plates, and the fluorescence of FITC-OVA was measured at an emission wavelength of 530 nm with an excitation wavelength of 480 nm, using a fluorometric microplate reader (Infinite-200Pro M-Plex, Tecan Japan Co., Ltd., Kanagawa, Japan). The protein content of the lysate was determined by a Bradford assay using BSA as a standard. Absorbance was measured using the microplate reader at 570 nm. Uptake of FITC-OVA was indicated as µg per mg protein.

### 2.5. Animals

Animal care and experimental procedures were performed in accordance with the Guidelines for Animal Experimentation of Nagasaki University with approval from the Institutional Animal Care and Use Committee. Male C57BL/6N mice (5 weeks old) were purchased from Japan SLC (Shizuoka, Japan). After being transported, the mice were allowed to acclimate to their new environment for ≥1 day before the experiments. Pulmonary administration (40 µL) was performed by using a tongue depressor with light by spontaneous respiration in mice anesthetized by inhalation of isoflurane (Horiguchi et al., 2015).

### 2.6. Lung accumulation of complex

In order to examine the accumulation of OVA, the 40 µg Alexa647-OVA and the complex containing 40 µg Alexa647-OVA at a volume of 40 µL per mouse were administrated into mice by pulmonary route. Six days after the administration, mice were sacrificed and the lungs were dissected. The fluorescent intensity of Alexa647-OVA in the mouse lung was observed with a Xenogen IVIS Lumina System coupled with Living Image software for data acquisition (Xenogen, Co, Almeda, CA, USA). After the observation, those lungs were homogenized with lysis buffer and homogenates were centrifuged at 15,000 rpm (Kubota-3500, Kubota Corporation, Tokyo, Japan) for 5 min and the fluorescence of Alexa647 in those supernatants were determined with microplate leader at an excitation and an emission wavelength of 640 and 670 nm, respectively.

### 2.7. Immunization

Mice were immunized with a 5% glucose solution, 40 µg OVA, empty complex of 8 µg BK and 8 µg γ-PGA (vehicle) and OVA/BK/γ-PGA complex containing 40 µg OVA by pulmonary administration, 4 times weekly. Two weeks after the last immunization, bronchoalveolar lavage fluid (BALF) and serum were obtained. The BALF and serum were used for enzyme-linked immunosorbent assay (ELISA) assays.

### 2.8. Determination of OVA-specific antibodies induction

For OVA coating, 100 µL of OVA solution (10 µg/mL, in 1 M sodium hydrogen carbonate) was added to each well of the ELISA plates (Thermo Fisher Scientific Inc., Waltham, MA, USA) and incubated for overnight at 4 °C. The plates were washed three times with phosphate-buffered saline containing 0.05% Tween-20 (PBST), 200 µL of blocking reagent N 102 (Nichiyu, Co., Ltd., Tokyo, Japan) added to block nonspecific binding and then incubated for 6 h at 4 °C. The plates were washed two times with PBST. Then, 100 µL aliquots of 1000-fold diluted serum and undiluted BALF samples were added to each well and incubated overnight at 4 °C. After five times washing with PBST, 100 µL each of horseradish peroxidase (HRP) – conjugated goat anti-mouse IgG, IgA, IgM, IgE, IgG1, IgG2a, IgG2b and IgG3 (1:10,000) (Abcam, Cambridge, UK) – were added to each well and incubated at room temperature for 1 h and then washed five times with PBST. TMB One solution (Promega, WI, USA) was used and prepared according to the manufacturer’s instructions. The reaction was then stopped at 15 min by the addition of 1 N hydrochloric acid. Absorbance was read at 450 nm by using a microplate reader.

### 2.9. *In vivo* toxicity of OVA/BK/γ-PGA complex

OVA and OVA/BK/γ-PGA complex were administered into mice by the pulmonary route. BALF was obtained 3 and 24 h after administration from mice. Twenty-four hours after administration, the lung was also dissected. Lactate dehydrogenase (LDH) activity in the BALF was measured using QuantiChrom™ Lactate Dehydrogenase Kit (BioAssay Systems, CA, USA) according to the manufactory’s instruction. The lung samples were fixed in a 4% paraformaldehyde phosphate buffer solution. The sectioning and haematoxylin-eosin (HE) staining were entrusted to GenoStaff (Tokyo, Japan). HE-stained sections of the lung were observed by microscopy at 20 × magnification.

### 2.10. Statistical analysis

The statistical significance of differences between the two groups was assessed by performing Student’s *t*-test. Multiple comparisons among the groups were performed by performing Tukey’s test. *p*-Values < .05 was considered to be indicative of statistical significance.

## 3. Results

### 3.1. Physicochemical properties of the complex

We constructed an anionic OVA/BK/γ-PGA complex at a weight ratio of 1:0.2:0.2. The γ-PGA-coated complex had a particle size of approximately 105.4 nm and a ζ-potential of approximately −35.5 mV.

### 3.2. Cellular uptake of the complex

**Figure 1.**
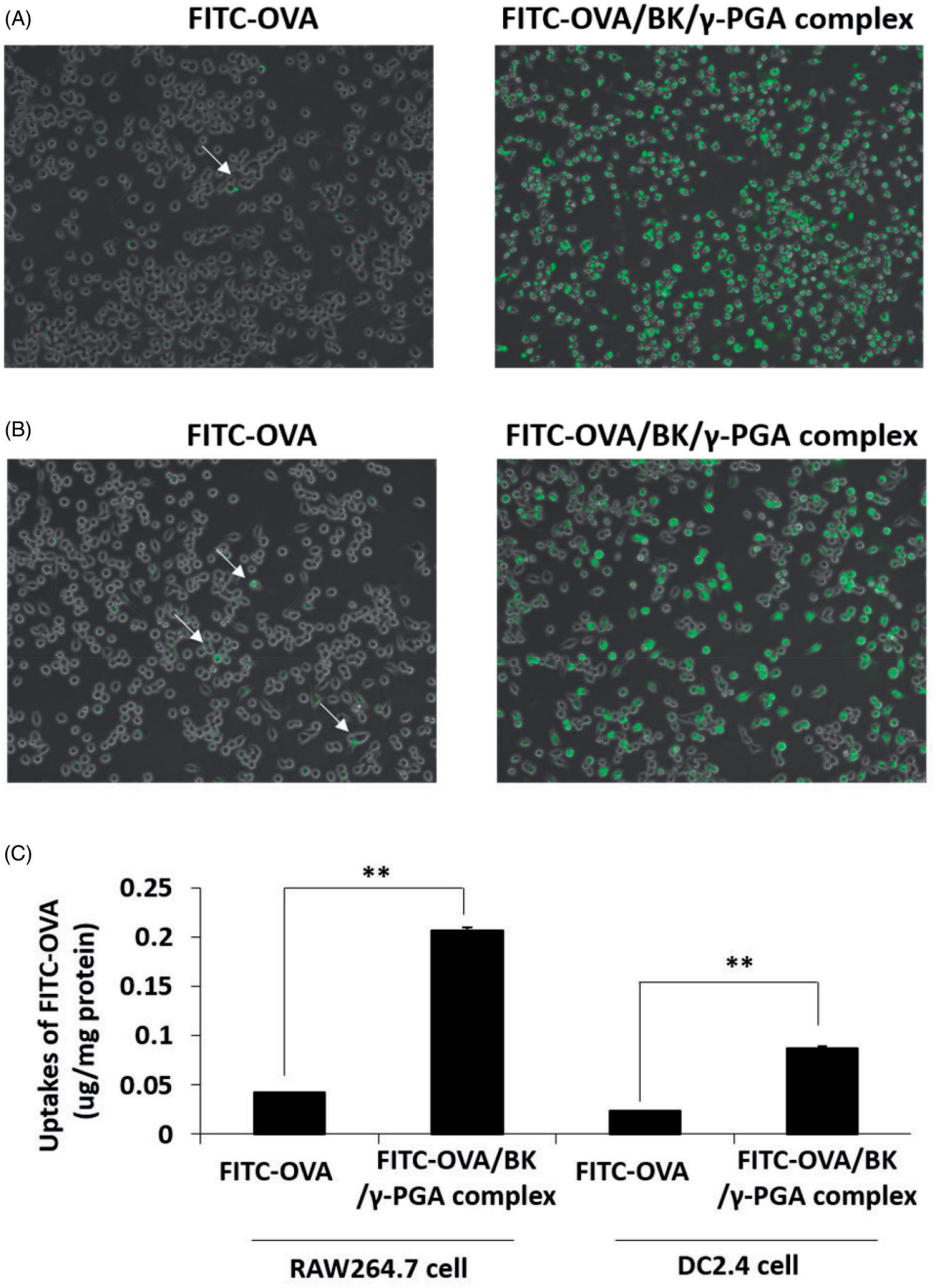
Cellular uptake of the FITC-OVA and FITC-OVA/BK/γ-PGA complex. RAW264.7 cells (A) and DC2.4 cells (B) were incubated with FITC-OVA and the FITC-OVA/BK/γ-PGA complex. Fluorescent images were acquired by performing fluorescence microscopy at 2 h and cellular uptakes of FITC-OVA were quatified (C). Each value represents the mean ± S.E. (*n* = 4). **p < .01.

RAW264.7 cells and DC2.4 cells were treated with FITC-OVA and FITC-OVA/BK/γ-PGA complex, and the cellular uptake of FITC-OVA was visualized, as shown in Figure 1. FITC-OVA/BK/γ-PGA complex was highly taken by the RAW264.7 cells and DC2.4 cells, and strong green fluorescence of FITC-OVA was observed (Figure 1(A,B), respectively). At the same time, little amounts of FITC-OVA were observed in both cells treated with FITC-OVA.

Cellular uptakes of FITC-OVA were quantified in the RAW264.7 cells and DC2.4 cells (Figure 1(C)). FITC-OVA/BK/γ-PGA complex showed significantly higher uptake than the FITC-OVA in both cells (*p* < .01).

### 3.3. Lung accumulation of Alexa647-OVA after pulmonary administration

**Figure 2.**
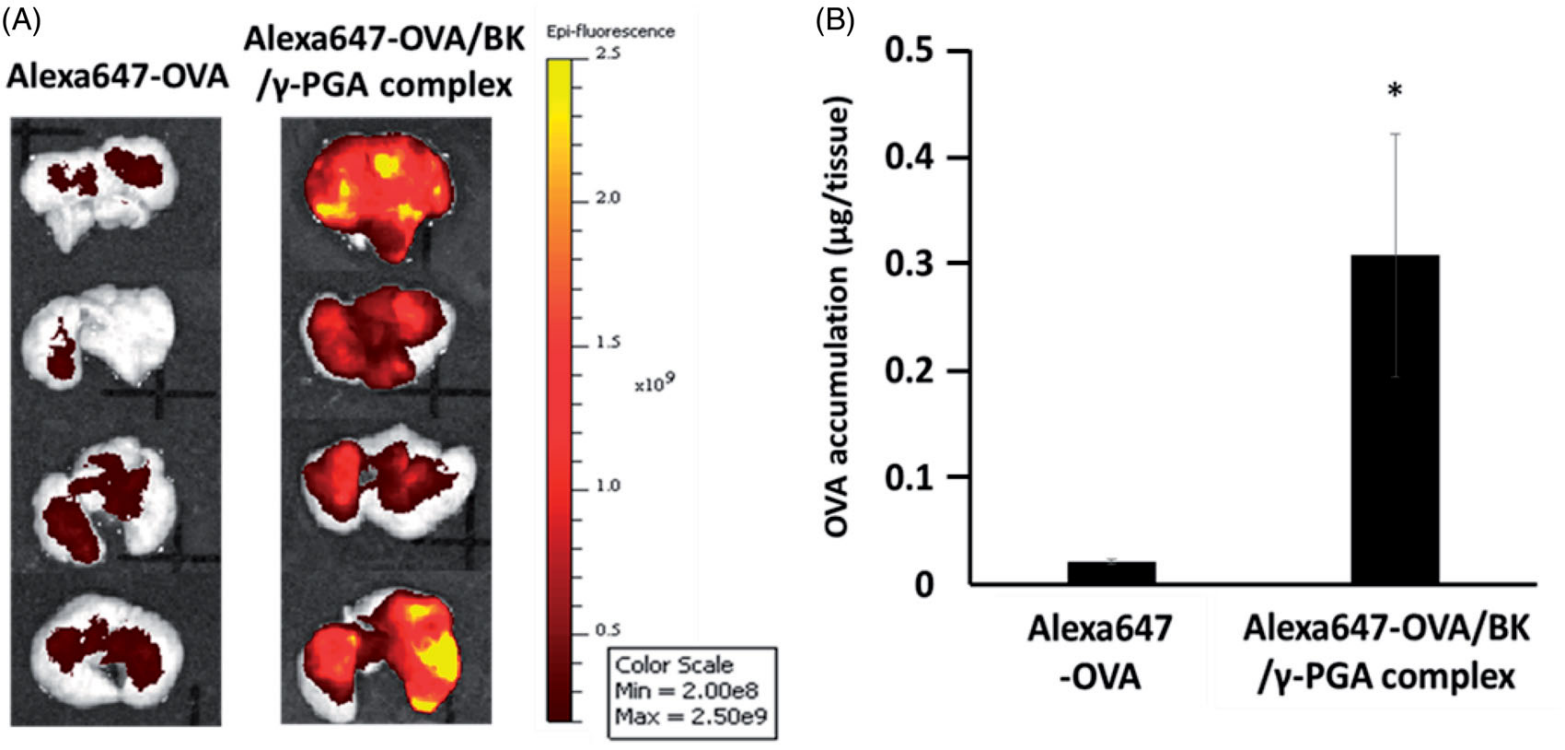
Lung accumulation of Alexa647-OVA after pulmonary administration. Mice were administered Alexa647-OVA or the Alexa647-OVA/BK/γ-PGA complex. At 6 days after pulmonary administration, the mice were sacrificed and their lungs dissected. Lung accumulation of Alexa647-OVA was visualized by using a Xenogen IVIS Lumina System (A) and quantified by using a microplate reader (B). Each value represents the mean ± S.E. (*n* = 4). *p < .05 vs. Alexa647-OVA.

Alexa647-OVA and Alexa647-OVA/BK/γ-PGA complex were administered into mice by the pulmonary route to determine the lung accumulation of Alexa647-OVA as antigen. At 6 days post-administration, the lungs were dissected, and their fluorescence intensity was visualized by using a Xenogen IVIS Lumina System. An *ex-vivo* fluorescent image is shown in Figure 2(A). High fluorescence intensity was observed throughout the whole lung at 6 days after pulmonary administration, although there was no fluorescence intensity in the spleen, heart, kidney, and liver (data not shown). Lung accumulation was higher for the Alexa647-OVA/BK/γ-PGA complex than for Alexa647-OVA on day 6. As shown in Figure 2(B), lung accumulation was significantly higher for the Alexa647-OVA/BK/γ-PGA complex than for Alexa647-OVA (*p* < .05).

### 3.4. OVA-specific antibody in serum after pulmonary administration of the complex

**Figure 3.**
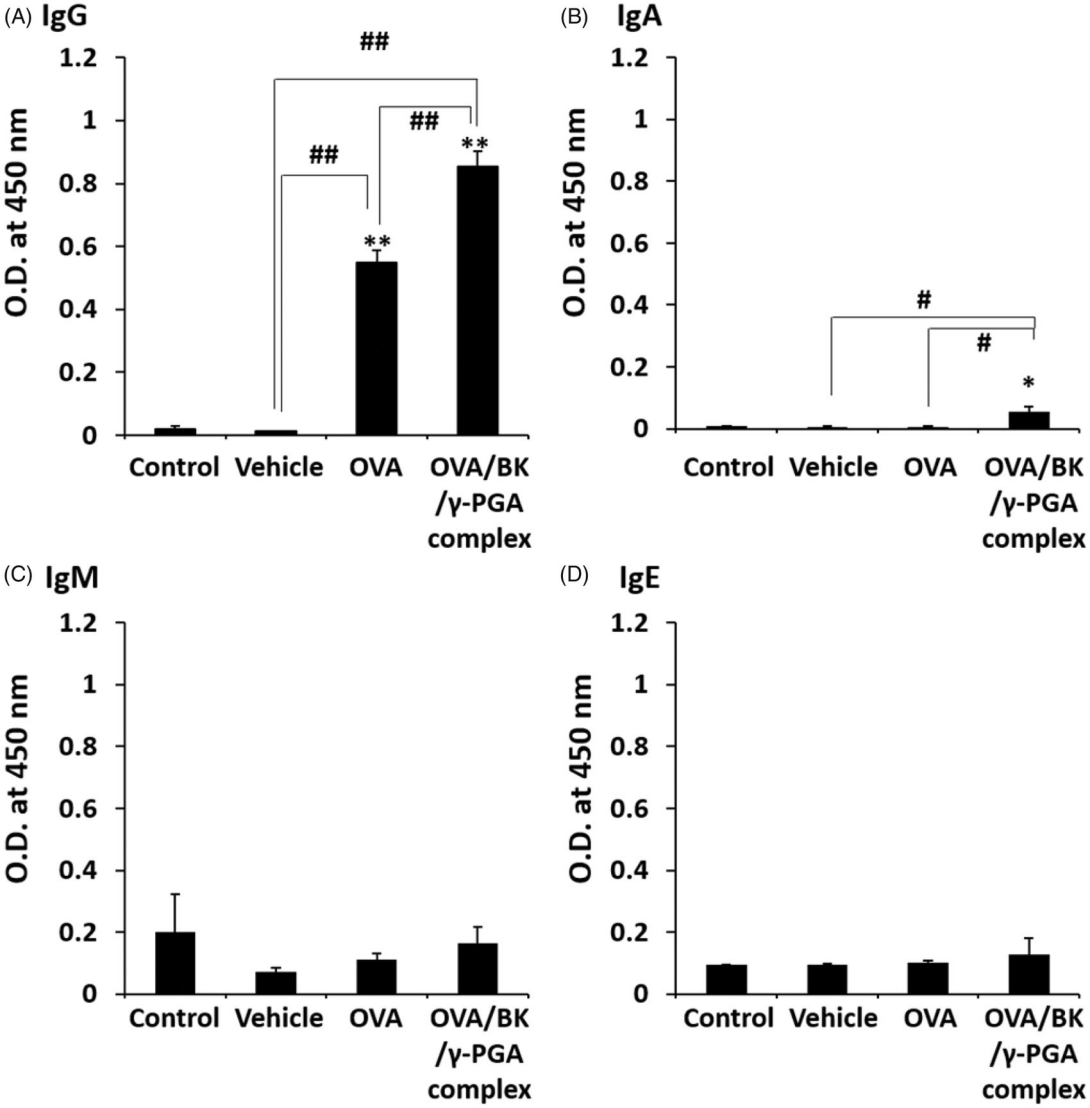
OVA-specific antibody in serum after pulmonary administration of the complex. Mice were treated four times with vehicle, OVA and the OVA/BK/γ-PGA complex weekly by pulmonary administration. Two weeks after the last administration, the mice were sacrificed, and serum samples were collected to measure OVA-specific IgG (A), IgA (B), IgM (C) and IgE (D) by ELISA. The 5% glucose solution was used as the control. Each value represents the mean ± S.E. (*n* = 4–5). *p < .05, **p < .01 vs. control, #*p* < .05, ##*p* < .01.

A 5% glucose solution, BK/γ-PGA complex (vehicle), OVA, and OVA/BK/γ-PGA complex were administered by the pulmonary route into mice four times, and then serum OVA-specific IgG, IgA, IgM and IgE were determined by ELISA as shown in Figure 3. Pulmonary administration of OVA and the OVA/BK/γ-PGA complex increased the IgG levels in mice, although OVA-specific IgG was not detected in mice administered 5% glucose and vehicle. Furthermore, IgG and IgA production were significantly higher after administration of the OVA/BK/γ-PGA complex than after administration of OVA (*p* < .05 or .01). On the other hand, OVA-specific IgM and IgE were not induced by OVA or the OVA/BK/γ-PGA complex.

### 3.5. OVA-specific antibody in BALF after pulmonary administration of the complex

**Figure 4.**
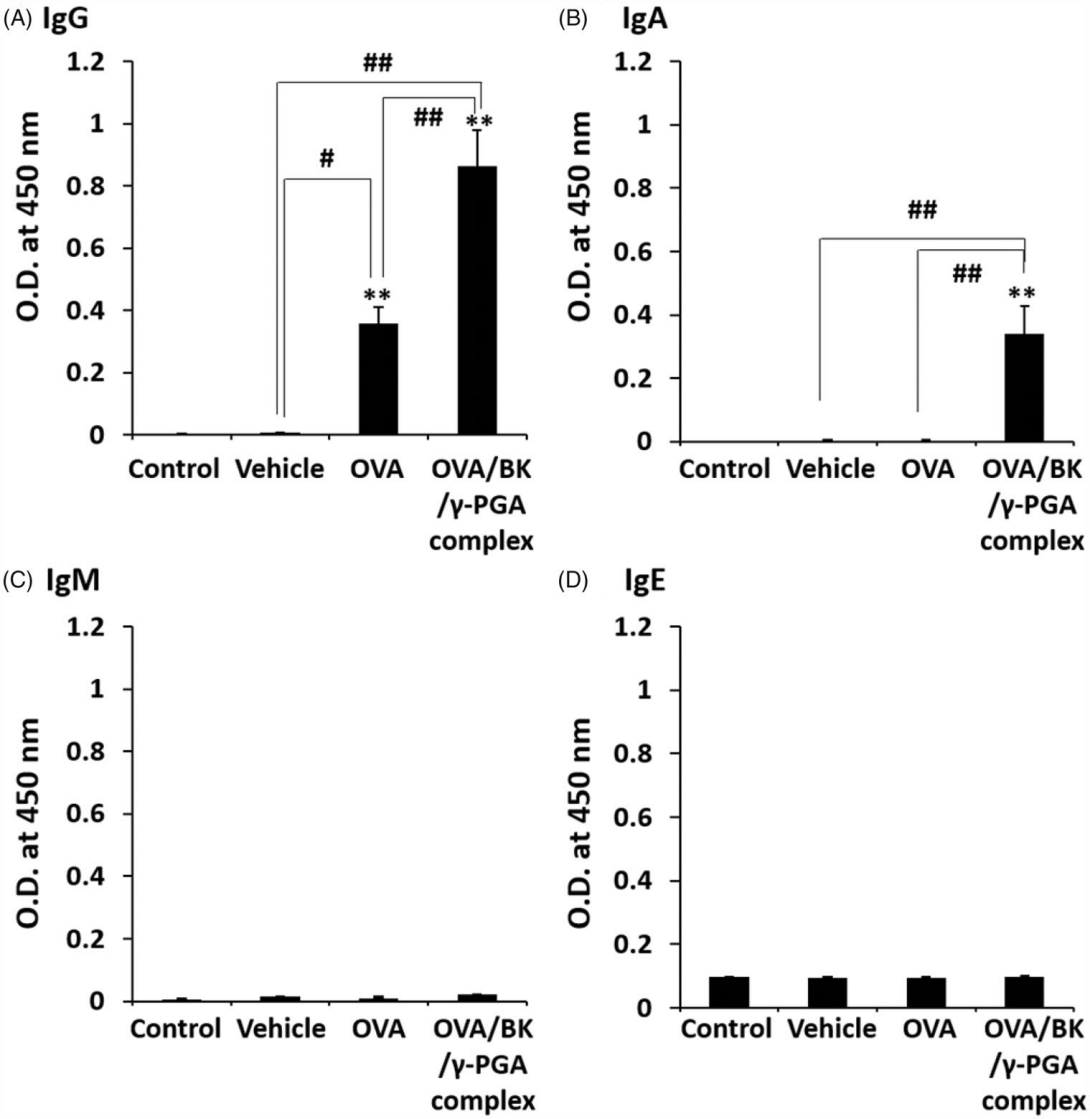
OVA-specific antibody in BALF after pulmonary administration of the complex. Mice were treated four times with vehicle, OVA and the OVA/BK/γ-PGA complex weekly by pulmonary administration. Two weeks after the last administration, the mice were sacrificed, and BALF samples were collected to measure OVA-specific IgG (A), IgA (B), IgM (C) and IgE (D) by ELISA. The 5% glucose solution was used as the control. Each value represents the mean ± S.E. (*n* = 4–5). **p < .01 vs. control, #*p* < .05, ##*p* < .01.

OVA-specific IgG, IgA, IgM and IgE in the BALF were also determined, as shown in Figure 4. The level of IgG was significantly increased by OVA relative to the levels after administration of the control and vehicle (*p* < .05 or .01). Nevertheless, the OVA/BK/γ-PGA complex significantly increased not only IgG but also IgA in the BALF (*p* < .01). However, OVA-specific IgM and IgE were not induced by OVA or the OVA/BK/γ-PGA complex.

### 3.6. IgG subtypes in serum after pulmonary administration of the complex

**Figure 5.**
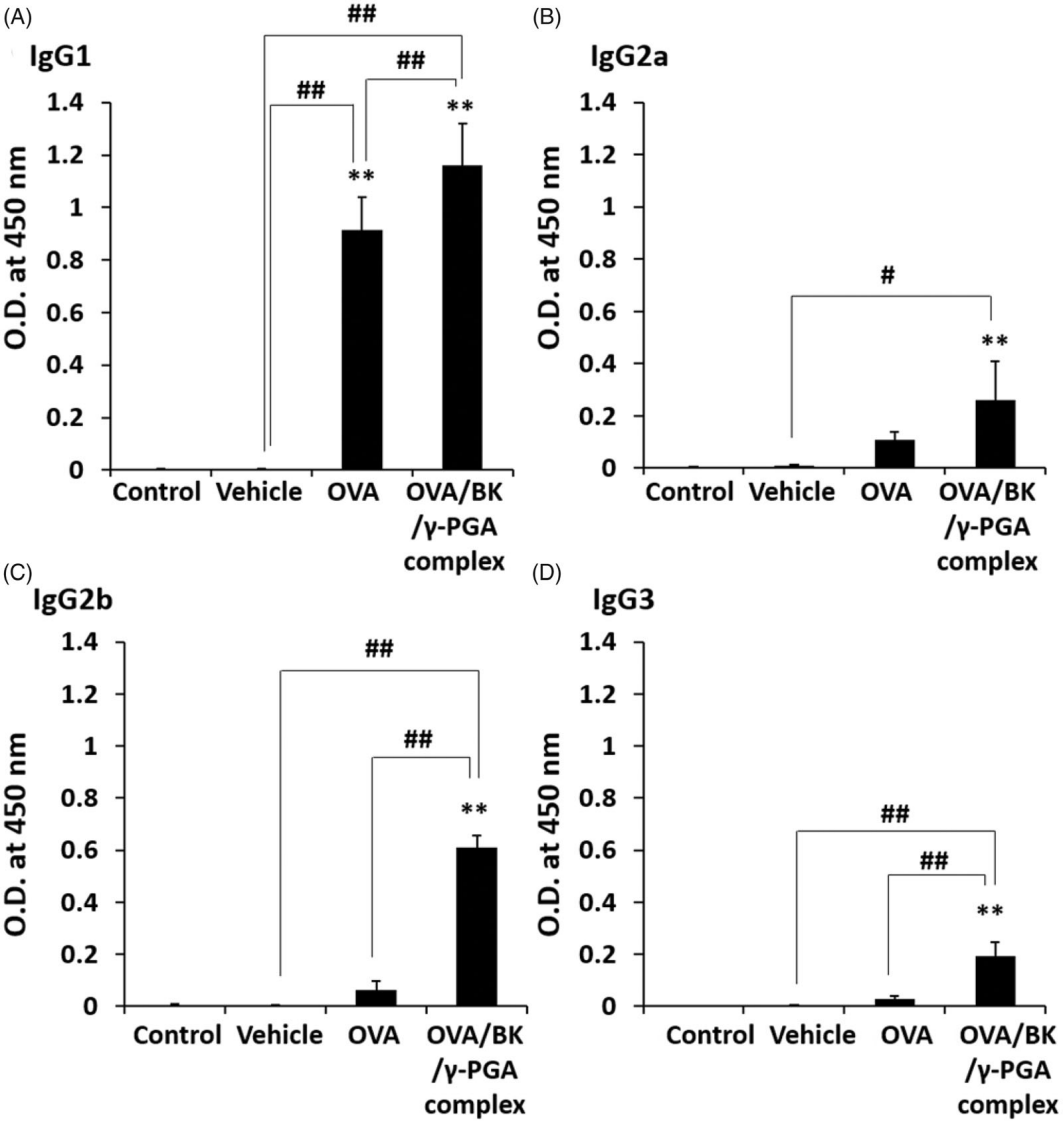
IgG subtypes in serum after pulmonary administration of the complex. Mice were treated four times with vehicle, OVA and the OVA/BK/γ-PGA complex weekly by pulmonary administration. Two weeks after the last administration, the mice were sacrificed, and blood samples were collected to measure OVA-specific IgG1 (A), IgG2a (B), IgG2b (C) and IgG3 (D) in the serum by ELISA. The 5% glucose solution was used as the control. Each value represents the mean ± S.E. (*n* = 4–5). **p < .01 vs. control, #*p* < .05, ##*p* < .01.

Induction effects of OVA-specific IgG subtypes, such as IgG1, IgG2a, IgG2b and IgG3 in the serum were determined as shown in Figure 5. Compared with the control and vehicle, OVA significantly increased only IgG1 (*p* < .01). The antibody levels of all subtypes induced by the OVA/BK/γ-PGA complex were significantly higher than the levels induced by the control and vehicle (*p* < .05 or .01).

### 3.7. IgG subtypes in BALF after pulmonary administration of the complex

**Figure 6.**
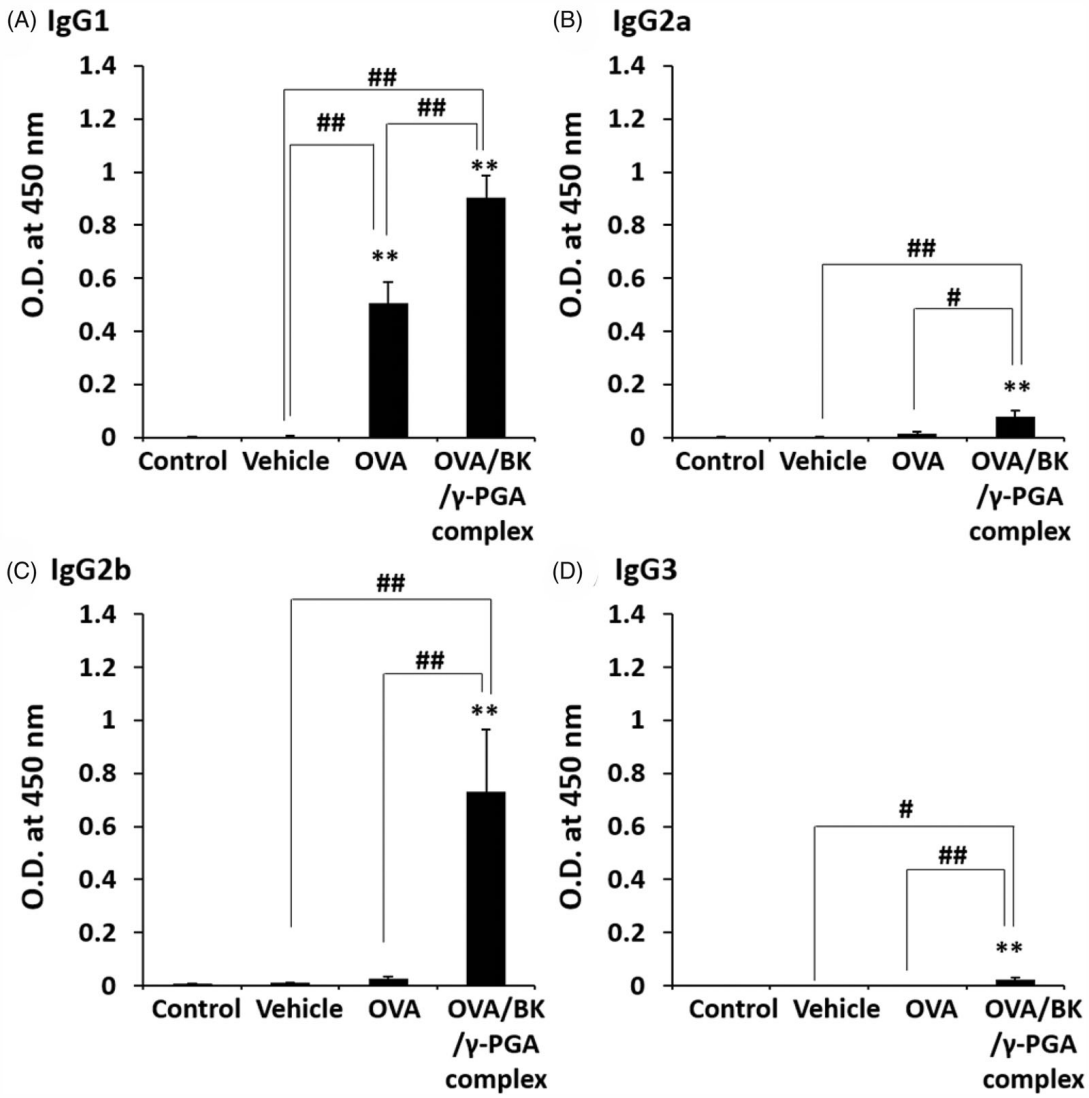
IgG subtypes in BALF after pulmonary administration of the complex. Mice were treated four times with vehicle, OVA and the OVA/BK/γ-PGA complex weekly by pulmonary administration. Two weeks after the last administration, the mice were sacrificed, and BALF samples were collected to measure OVA-specific IgG1 (A), IgG2a (B), IgG2b (C) and IgG3 (D) by ELISA. The 5% glucose solution was used as the control. Each value represents the mean ± S.E. (*n* = 4–5). **p < .01 vs. control, #*p* < .05, ##*p* < .01.

OVA-specific IgG subtypes, such as IgG1, IgG2a, IgG2b and IgG3 in the BALF were also determined, as shown in Figure 6. OVA significantly increased only IgG1 levels relative to those induced by the control and vehicle (*p* < .01). Compared with the control, vehicle, and OVA, the OVA/BK/γ-PGA complex induced significantly higher antibody levels of all subtypes (*p* < .05 or .01).

### 3.8. *In vivo* toxicity of OVA/BK/γ-PGA complex

**Figure 7.**
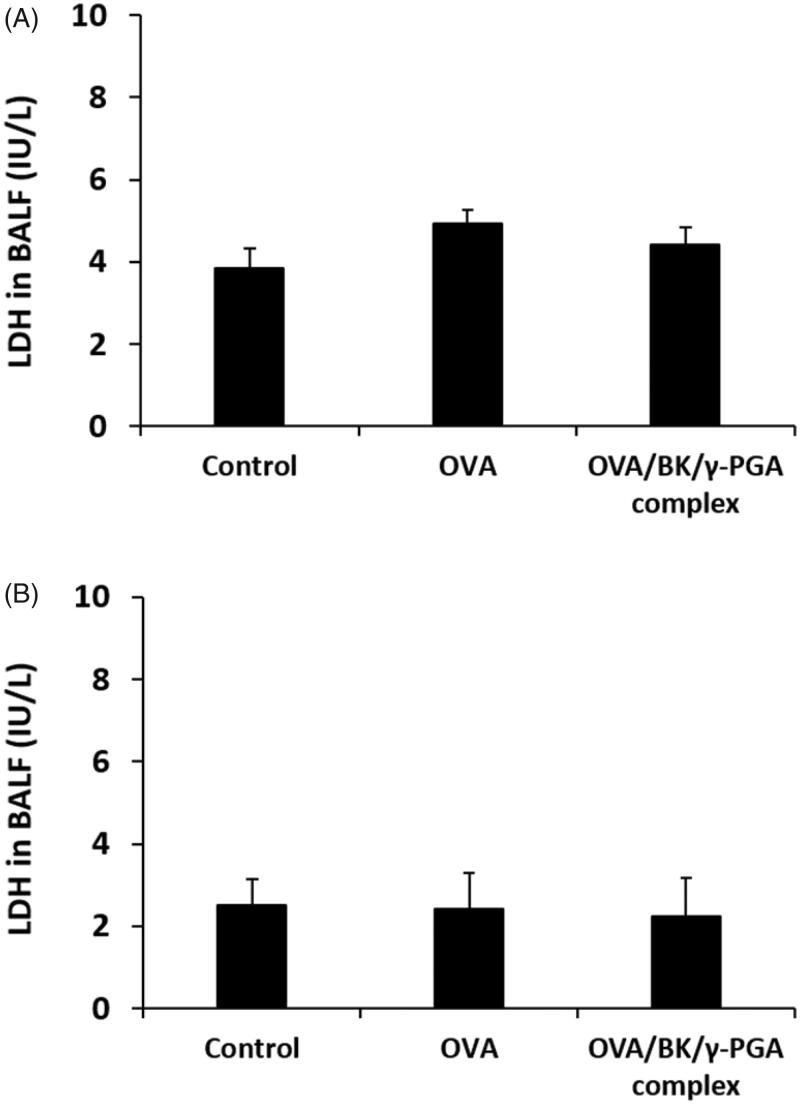
LDH level in the BALF after pulmonary administration of 5% glucose, OVA and the OVA/BK/γ-PGA complex. Five percent glucose solution, OVA and the OVA/BK/γ-PGA complex were administered to mice. Three and twenty-four hours after administration (A and B, respectively), BALF was taken from the mice and LDH activity in the BALF was determined. Each value represents the mean ± S.E. (*n* = 4).

Mice were administered 5% glucose solution, OVA, and the OVA/BK/γ-PGA complex. LDH activity in the BALF was determined 3 or 24 h after administration (Figure 7). Administration of OVA and OVA/BK/γ-PGA complex had little effect on the LDH levels in the BALF.

**Figure 8.**
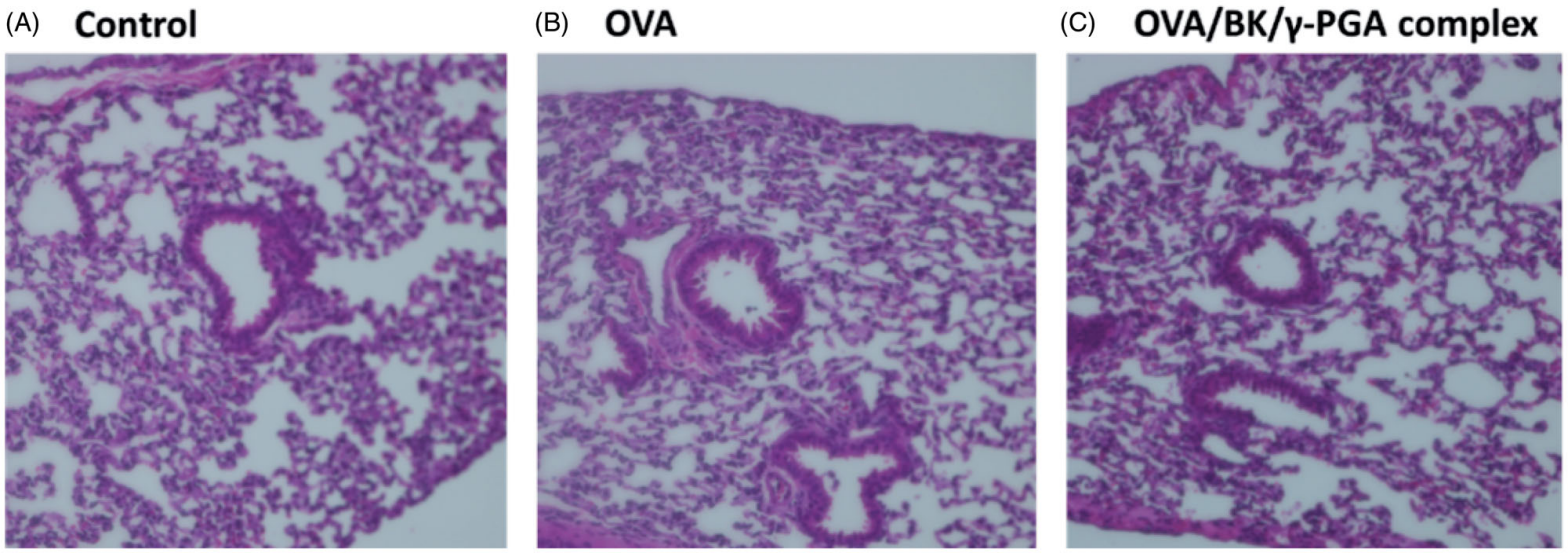
Lung micrograph after pulmonary administration of 5% glucose (A), OVA (B) and the OVA/BK/γ-PGA complex (C). Five percent glucose solution, OVA and the OVA/BK/γ-PGA complex were administered to mice. Twenty-four hours after administration, the mice were sacrificed and their lungs dissected. HE-stained sections of the lung were observed by microscopy at 20× magnification.

Twenty-four hours after administration, lungs were dissected from those mice for histological analysis. HE-stained lung sections are shown in Figure 8. No histological abnormalities were observed in the mice treated with OVA and the OVA/BK/γ-PGA complex.

## 4. Discussion

The lung controls breathing and is exposed to many pathogens, such as viruses and bacteria that cause respiratory infections. Those pathogens infect through the pulmonary mucosal membrane, so mucosal immunity must be induced to protect against respiratory infections. To prevent respiratory infections, mucosal immunity has an important role; however, intradermal and intramuscular administration of the vaccine cannot strongly stimulate mucosal immunity (Ito et al., 2003; Amorij et al., 2007). The mucosal immune system that induces secretory IgA is developed on the mucosal surface. Several APCs, such as dendritic cells and macrophages, have been reported to be located in the mucosal surface (Kopf et al., 2015). Furthermore, bronchus-associated lymphoid tissue (BALT) is found in the bronchiolar mucosa and is a lymphoid follicle that has a central role in respiratory tract mucosal immunity (Bienenstock, 1980). Pulmonary administration of the vaccine is expected to stimulate the mucosal immune system effectively. Therefore, we constructed the OVA/BK/γ-PGA complex to assess its usefulness as a vaccine administered via the pulmonary route.

Figure 4 shows the BALF concentration of OVA-specific antibodies after pulmonary administration of OVA and the OVA/BK/γ-PGA complex. Pulmonary administration of OVA increased IgG but had little effect on IgA in BALF. On the other hand, the OVA/BK/γ-PGA complex significantly induced IgA secretion in the BALF. Furthermore, serum IgG induction was significantly higher after administration of the OVA/BK/γ-PGA complex than after administration of OVA (Figure 3). Serum IgG is important for preventing aggravation of any infections that occur (Huber et al., 2006; Schroeder & Cavacini, 2010). The results indicate that the OVA/BK/γ-PGA complex could prevent respiratory infections by high mucosal IgA induction and aggravation of respiratory infection by high serum IgG induction. Furthermore, IgE induction by the OVA/BK/γ-PGA complex was not observed, and this result indicated little risk of allergic reaction by the OVA/BK/γ-PGA complex.

The OVA/BK/γ-PGA complex could also induce not only IgG1 and IgG2b but also IgG2a and IgG3 (Figure 5). IgG1 and IgG2b are known as Th2-type immunoglobulins that mediate the humoral immune response, and IgG2a and IgG3 are known as Th1-type immunoglobulins that mediate the cellular immune response (Firacative et al., 2018). Therefore, the OVA/BK/γ-PGA complex could induce both the humoral and cellular immune responses. These results suggest that this system could be useful for vaccines against infections as well as cancers. The preventive effect, antigen-induced Th1- and Th2-type cytokine responses in inducible BALT, Treg population, and IL-10 induction should be assessed using real antigens from infections or cancers in future studies.

The strong induction by the OVA/BK/γ-PGA complex of the immune response must be due to the high retention of the complex in the lung and effective delivery of OVA into the APCs in the pulmonary mucosa. Our results confirmed this by showing that 6 days after administration, the Alexa647-OVA/BK/γ-PGA complex still remained in the lung and showed significantly higher fluorescence than Alexa647-OVA (Figure 2). The complex formation might prevent diffusion of OVA and protect OVA from degradation by proteases in the pulmonary mucosa. Furthermore, the OVA/BK/γ-PGA complex was able to effectively deliver OVA into RAW264.7 macrophage cells and DC2.4 dendritic cells (Figure 1). Those results indicated that the OVA/BK/γ-PGA complex would be taken up by alveolar APCs and induce a high immune response after pulmonary administration. It has been reported that γ-PGA–coated nanoparticles were taken up by the gamma-glutamyl transpeptidase-mediated endocytotic pathway (Du et al., 2015). We also confirmed the γ-PGA–coated nanoparticles were taken up by the γ-PGA specific endocytotic pathway (Kurosaki et al., 2009). The OVA/BK/γ-PGA complex might be taken up by APCs in the lung through the same mechanisms. However, it is still not clear that which cells are responsible for OVA-uptake and subsequent antigen presentation in the induction of specific immunity. Further studies about detailed immune induction mechanisms should be performed in the future.

Many adjuvants have been developed to enhance the effectiveness of vaccines; however, adjuvants have been reported to cause inflammatory reactions at the injection site (Tamura et al., 1988; McKee et al., 2007). Therefore, administration of adjuvants to the lung might cause severe side effects, such as pneumonia. After pulmonary administration of the OVA/BK/γ-PGA complex, LDH levels in the BALF were not increased (Figure 7) and histological abnormalities were not observed in the HE-stained section (Figure 8). Reportedly, γ-PGA is a biocompatible and biodegradable polymer that does not show immuno-inflammatory reactions (Prodhomme et al., 2003; Ye et al., 2006). BK is a safe quaternary ammonium compound that has been widely used clinically as an antimicrobial additive (Marple et al., 2004). These reports also support the safety of the OVA/BK/γ-PGA complex. On the other hand, higher LDH levels were observed at 3 h after the pulmonary administration than at 24 h, even in the control group. Those results are indicating that a slight irritation was caused by pulmonary administration. Further safety studies are needed before clinical application of the OVA/BK/γ-PGA complex.

In the present study, we demonstrated high mucosal immune system induction by a novel vaccine delivery vector constructed with an antigen protein, BK, and γ-PGA after pulmonary administration. The system can be used for vaccines against various respiratory infections.
